# Improving Reproductive Health Communication Between Providers and Women Affected by Homelessness and Substance Use in San Francisco: Results from a Community-Informed Workshop

**DOI:** 10.1007/s10995-023-03671-y

**Published:** 2023-05-19

**Authors:** Erin E. Wingo, Sara J. Newmann, Deborah E. Borne, Brad J. Shapiro, Dominika L. Seidman

**Affiliations:** 1 Person-Centered Reproductive Health Program (PCRHP), Department of Family and Community Medicine, University of California, San Francisco, San Francisco, CA USA; 2 Department of Obstetrics, Gynecology & Reproductive Services, University of California, San Francisco, San Francisco, CA USA; 3 Transitions Division, San Francisco Health Network, San Francisco Department of Public Health, San Francisco, CA USA; 4 Department of Psychiatry, University of California, San Francisco, San Francisco, CA USA

**Keywords:** Substance use disorder, Homelessness, Reproductive health services, Professional education

## Abstract

**Objectives:**

Many cisgender women affected by homelessness and substance use desire pregnancy and parenthood. Provider discomfort with patient-centered counseling about reproductive choices and supporting reproductive decisions of these women poses barriers to reproductive healthcare access.

**Methods:**

We used participatory research methods to develop a half-day workshop for San Francisco-based medical and social service providers to improve reproductive counseling of women experiencing homelessness and/or who use substances. Guided by a stakeholder group comprising cisgender women with lived experience and providers, goals of the workshop included increasing provider empathy, advancing patient-centered reproductive health communication, and eliminating extraneous questions in care settings that perpetuate stigma. We used pre/post surveys to evaluate acceptability and effects of the workshop on participants’ attitudes and confidence in providing reproductive health counseling. We repeated surveys one month post-event to investigate lasting effects.

**Results:**

Forty-two San Francisco-based medical and social service providers participated in the workshop. Compared to pre-test, post-test scores indicated reduced biases about: childbearing among unhoused women (*p* < 0.01), parenting intentions of pregnant women using substances (*p* = 0.03), and women not using contraception while using substances (*p* < 0.01). Participants also expressed increased confidence in how and when to discuss reproductive aspirations (*p* < 0.01) with clients. At one month, 90% of respondents reported the workshop was somewhat or very beneficial to their work, and 65% reported increased awareness of personal biases when working with this patient population.

**Conclusions for Practice:**

A half-day workshop increased provider empathy and improved provider confidence in reproductive health counseling of women affected by homelessness and substance use.

## Significance Statement

In the setting of significant trauma histories, untreated mental illness, and trauma directly related to housing instability, women experiencing homelessness (WEH) in the United States are also frequently affected by substance use. Facing stigma, judgment, mistreatment, and under-trained providers, WEH using substances often have limited access to reproductive health services and experience poor reproductive health outcomes. A half-day provider training, designed in collaboration with a stakeholder group of women with lived experience and community partners in San Francisco, CA increased provider empathy and improved provider confidence in reproductive health counseling of women affected by homelessness and substance use.

## Introduction

Cisgender women comprise a sizable share of people experiencing homelessness, with unsheltered homelessness growing by 5% among women and girls between 2020 and 2022 (U.S. Department of Housing & Urban Development, 2022), and face immense health and healthcare inequities. These include disproportionate rates of poor reproductive and pregnancy outcomes (Clark et al., 2019; DiTosto et al., 2021; St Martin et al., 2021) and inadequate and inconsistent access to reproductive health care (Corey et al., 2020; Dasari et al., 2016; Lewis et al., 2003; McGeough et al., 2020; Schmidt et al., 2023; Teruya et al., 2010). A number of interconnected individual and structural challenges contribute to these inequities, including lack of social support and stability, difficulty accessing and navigating unwelcoming healthcare and social service systems, fear of child welfare involvement and removal of children, and poor treatment in healthcare settings (Allen & Vottero, 2020; Frazer et al., 2019; Gelberg et al., 2004; McGeough et al., 2020; Schmidt et al., 2023).

Substance use disorders disproportionately affect people experiencing homelessness for myriad reasons. People experiencing homelessness may manage the trauma of homelessness with substance use, and access to substance use disorder treatment is more challenging in the setting of unstable housing (Frazer et al., 2019; Magwood et al., 2020). In addition, people experiencing homelessness have disproportionately experienced childhood trauma, which is associated with future substance use (Khoury et al., 2010; Liu et al., 2021; Magwood et al., 2020). Thus, women and other people capable of pregnancy experiencing homelessness may present to a diverse range of low-barrier healthcare settings, including homeless health outreach programs, drop-in centers, and substance use treatment facilities, with the desire to address a wide range of health issues including preconception care, pregnancy care, and pregnancy prevention.

Given complex access challenges, intentional, patient-centered interaction in these settings is critical to facilitate reproductive healthcare engagement. However, providers may have unchecked assumptions about reproductive aspirations and capabilities of people affected by homelessness and substance use that affect if and how they engage with women and other individuals about their reproductive goals and needs. Women experiencing homelessness and with substance use disorders have reported feeling dehumanized, disrespected and judged during reproductive health care encounters; receiving substandard care; feeling unable to advocate for themselves; and being coerced into contraception use (Begun et al., 2019; Dasari et al., 2016; Frazer et al., 2019; Kennedy et al., 2014; MacAfee et al., 2020; Sznajder-Murray & Slesnick, 2011; Terplan et al., 2015). Demeaning treatment from providers and a lack of agency in clinical spaces decreases trust and rapport (Azarmehr et al., 2018; Bloom et al., 2004). Thus, unsurprisingly, provider attitudes toward and poor treatment of women affected by homelessness and substance use has served as a barrier to entry and retention in care, in particular contraceptive care and antenatal care (Allen & Vottero, 2020; Begun et al., 2019; Kennedy et al., 2014; MacAfee et al., 2020; Sznajder-Murray & Slesnick, 2011).

Some limited evidence suggests that provider and staff training, promoting critical self-awareness of biases and attitudes, can improve care engagement of people experiencing homelessness (Aparicio et al., 2019; Rew et al., 2008). However, interventions addressing provider communication and biases toward the reproductive health and well-being of cisgender women and other people capable of pregnancy affected by substance use and homelessness are rare, and few incorporate the perspectives of affected individuals themselves. Recent work aimed at decreasing racial disparities in pregnancy and birth outcomes uplifts the importance of centering the voices of the impacted populations in defining reproductive care priorities and development of robust and just healthcare systems (Altman et al., 2020; Franck et al., 2020). These lessons resonate in the context of women affected by homelessness and substance use, whose reproduction is frequently devalued and who are rarely invited to engage in the development of care priorities.

Motivated by the voiced needs of affected women and under the guidance of a stakeholder group including individuals with lived experience, we developed a workshop to improve provider-patient reproductive health communication and address provider attitudes that may result in enacted stigma and discrimination. We hosted the workshop for providers who cisgender women frequently contacted: homelessness services providers, reproductive health providers, and substance use treatment providers. Here, we describe (1) workshop development in partnership with a community advisory board of relevant stakeholders; (2) a quasi-experimental, single group pretest–posttest questionnaire with one-month-post intervention follow-up; and (3) qualitative interviews with study participants to understand workshop acceptability and effect on provider attitude among providers serving women affected by homelessness and substance use in San Francisco, CA.

## Methods

### Workshop Development Process

To guide the intervention development, we conducted a 6-month needs assessment with cisgender women experiencing homelessness and using substances in San Francisco and the providers that serve them. This assessment is described elsewhere (Schmidt et al., 2023). Briefly, we found a striking disconnect between patients’ and providers’ recommendations to improve reproductive services for affected individuals: while provider recommendations focused on improving access, patients focused on improving patient-provider communication and respectful care. Guided by this discordance, we developed a community-informed intervention to educate providers.

### Community-Driven Intervention Development

We used an iterative process to develop workshop content over six months, guided by semi-structured participatory discussions with a community stakeholder group. Group members (*n* = 10) were recruited to represent the experiences of people who had been pregnant while experiencing homelessness and/or using substance, and/or service providers in the areas of homelessness, substance use treatment, or reproductive health. The group convened for 2-h, monthly, and guided the workshop development process.

**Fig. 1:**
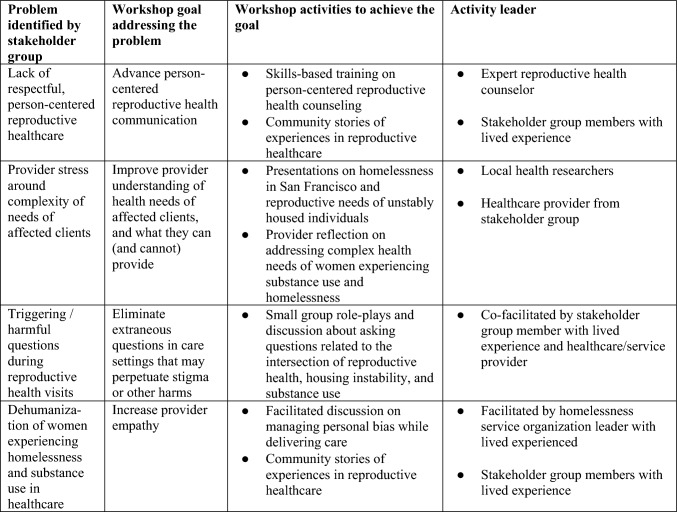
Development of a workshop on reproductive health communication with women experiencing homelessness (WEH) and substance use

Through facilitated discussions and generative activities, we identified group priorities. The project manager took notes at each stakeholder group meeting and incorporated feedback into workshop development. Prominent themes included lack of respect and empathy during reproductive health care, provider anxiety about the complexity of client needs, triggering and harmful questions employed in care settings, and an overarching feeling of dehumanization in healthcare spaces. Based on these topic areas, we drafted a workshop outline (Fig. 1). Stakeholder group members then guided the development of interactive sessions to be held at the workshop and received training in facilitation.

The stakeholder group agreed that community stories were a critical addition to workshop content. We recruited community members to share personal stories about their experiences accessing and receiving reproductive health care services. A peer story-telling expert used qualitative interviewing techniques to elicit stories and develop them into scripts to be delivered at the workshop. Final scripts were reviewed and approved by each community member who contributed the story.

### Workshop Structure

In consultation with the stakeholder group members and other key informants, we planned a four-hour workshop. Derived from priorities identified by the stakeholder group, goals of the workshop included: to increase provider empathy, advance patient-centered reproductive health communication, and eliminate extraneous questions in care settings that may perpetuate stigma or trauma (Fig. 1, column 2).

The workshop consisted of didactic, storytelling, and interactive sessions on managing personal biases and exploring intake and counseling approaches specific to women affected by homelessness and substance use (Fig. 1, column 3). Between each session, a member of the stakeholder group presented one of the scripted personal stories. Finally, participants received a description of and contact information for organizations represented in the room to facilitate knowledge of resources and motivate cross-agency collaboration.

### Workshop Participant Recruitment

We recruited workshop participants who work at the nexus of homelessness services, substance use, and reproductive health. We employed a purposive sampling strategy, sending invitations to contacts in health and social service city agencies, university-affiliated programs, and non-profit organizations across San Francisco. We asked participants to register for the workshop and reviewed the proportion of providers working in different areas who registered during the registration period, so that we could conduct targeted outreach to under-represented agencies.

### Study Design and Analysis

Our evaluation aimed to measure reaction, learning, and behavior change (Kirkpatrick, 1994). Participants completed questionnaires immediately before and after the workshop, providing responses about professional characteristics and pre-post attitude measures. Pre-post items were chosen to indicate degrees of bias toward reproductive aspirations of people experiencing homelessness or using substances and provider confidence on engaging around core topic areas with affected individuals. Items were measured on a 5-point Likert scale and adapted from existing measures (Fine et al., 2013; Goggin et al., 2018), and from themes identified in our local needs assessment. We also asked participants to complete a questionnaire one month after the workshop and invited participants to participate in semi-structured phone interviews about their workshop experience. Both the immediate- and one month-post questionnaires included short answer prompts as well as multiple-choice questions. We remunerated participants with a $20 gift card for completing the one-month follow-up survey and a $30 gift card for completing an in-depth interview.

We computed frequencies for all quantitative measures in Stata 14 (StataCorp LLC, College Station, TX). We calculated median scores and interquartile ranges for each pre/post-test measure of attitudes and confidence and used Wilcoxon sign tests to measure changes before and after the workshop and between directly post and one-month following the workshop.

Short answers and in-depth interviews were analyzed in Atlas.ti version 8 (Scientific Software Development GmbH, Berlin, Germany). Three authors developed a codebook based on a priori established domains. Codes were then applied by a single coder and refined based on emergent themes. Analysis was considered complete when no new themes emerged.

The evaluation protocol was reviewed and deemed exempt by the institutional review board of the University of California, San Francisco.

## Results

### Participant Professional Characteristics

Forty-two San Francisco-based medical and social service providers who work in reproductive health (70%), substance use (59%), and/or homelessness (80%) participated in the workshop in June 2019 (Table 1). Twenty-six percent were clinicians, (MDs or DOs), 29% were nurses, with additional representation from case managers and outreach workers, social workers, health services mangers, and health educators. Further professional characteristics are presented in Table 1.

**Table 1:** Workshop participant professional characteristics

	*N* = 42	%
Position		
Clinician (MD, DO)	11	26
Counselor/Health educator	1	2
Nurse	12	29
Health services manager/admin	4	10
Social worker	2	5
Case manager/outreach worker/navigator	3	7
Other	9	21
Organization type		
Community based organization	13	31
City government	14	33
County hospital or safety net clinic	12	29
Private organization	1	2
Other	2	5
Focus area*		
Homelessness	33	80
Substance use	24	59
Reproductive health	29	70
Length of time in current position**		
< 1 year	8	19
1–4 years	17	41
≥ 5 years	16	39
Length of time in field**		
< 1 year	3	7
1–2 years	6	15
3–5 years	7	17
≥ 6 years	25	61

*Participants could choose more than 1 focus area

**Missing data from 1 participant

### Pre-Post Assessment

Compared to pre-test, post-test scores indicated a decrease in negative feelings about childbearing among women experiencing homelessness (*p* < 0.01), parenting intentions of women using substances during pregnancy (*p* < 0.05), and women not using contraception while using substances (*p* < 0.001; Table 2). Regarding assumptions about contraceptive need, respondents more frequently disagreed with the following statement after the workshop: “people experiencing homelessness do not use contraception because they don’t know where to access it” (*p* < 0.01). Additionally, participants expressed increased confidence in how and when to discuss substance use (*p* = 0.01) and reproductive aspirations (*p* < 0.01) with clients. Finally, post-test results indicated a trend towards participants feeling more overwhelmed by the life complexity of women experiencing homelessness (*p* < 0.10), though this was not statistically significant.

**Table 2:** Pre- and post- workshop assessment of provider attitudes toward homelessness, substance use and pregnancy/parenting

	Pre-survey*Median (IQR)*	Post-survey *Median (IQR)*	^‡^
Healthcare providers should address the physical and social problems of patients	5.0 (5.0–5.0)	5.0 (4.0–5.0)	
I respect that my clients’ priorities may be more important to them than following my recommendations	5.0 (5.0–5.0)	5.0 (5.0–5.0)	
People should not have children when they are homeless	2.0 (1.0–3.0)	2.0 (1.0–2.0)	*
It is irresponsible for people who use substances to not use contraception	3.0 (2.0–3.0)	2.0 (1.0–2.0)	*
People who use substances while pregnant care little about themselves	2.0 (1.0–2.0)	1.0 (1.0–2.0)	
People who continue to use substances after they are pregnant care little about the baby	2.0 (1.0–2.0)	1.0 (1.0–2.0)	†
People experiencing homelessness don’t use contraception because they don’t know where to access it	3.0 (3.0–3.0)	2.0 (2.0–3.0)	*
I feel confident in how and when to discuss housing status with clients	4.0 (4.0–5.0)	4.0 (4.0–5.0)	
I feel confident in how and when to discuss substance use with clients	4.0 (3.0–5.0)	4.0 (4.0–5.0)	*
I feel confident in how and when to discuss reproductive health desires with people experiencing homelessness	4.0 (3.0–4.0)	4.0 (4.0–5.0)	*
I feel overwhelmed by the complexity of the problems that people experiencing homelessness have	3.0 (2.0–4.0)	4.0 (2.0–4.0)	†
I don’t ask my clients about reproductive health because I don’t know where to refer them for care	2.0 (1.0–2.0)	2.0 (1.0–3.0)	
I know what reproductive health services are available in the city for my clients who are homeless or using substances	4.0 (3.0–4.0)	4.0 (4.0–5.0)	
I know what social support services are available in the city for my clients who are homeless or using substances	4.0 (4.0–4.0)	4.0 (3.0–4.0)	

*1* = *Strongly disagree, 2* = *Disagree, 3* = *Unsure, 4* = *Agree, 5* = *Strongly agree*

*Significant at *p* < .05

†significant at *p* < .10

^‡^Significance determined by Wilcoxin sign tests comparing distribution of responses between baseline and immediate post-workshop survey responses (*n* = 42)

### Impact Survey Findings

Thirty participants (71% of workshop participants) completed the one-month follow up survey. Of those participants, ninety percent reported that the workshop was somewhat or very beneficial to their work. Many participants reported that they continued to have increased confidence in when and how to initiate conversations about reproductive health (64%), substance use (55%), and housing status (52%) with clients experiencing homelessness and/or using substances (Table 3). Sixty-six percent reported increased awareness of personal biases when working with women affected by homelessness or substance use.

**Table 3:** Self-reported confidence and knowledge about workshop skills at one-month follow-up survey

	Decreased *n* (%)	About the same *n* (%)	Increased *n* (%)
Confidence in when and how to initiate conversations about reproductive health with clients experiencing homelessness or with substance use disorders	–	10 (34)	19 (64)
Confidence in when and how to initiate conversations about substance use with clients experiencing homelessness	–	13 (45)	16 (55)
Confidence in when and how to initiate conversations about housing status with clients	–	14 (48)	15 (52)
Knowledge of resources available in San Francisco	–	15 (52)	14 (48)
Understanding of experiences of women experiencing homelessness engaging in reproductive health care	–	7 (24)	22 (76)
Awareness of personal bias when working with women experiencing homelessness or using substances	–	10 (34)	19 (66)

### Interview Findings

We conducted follow-up interviews with eight workshop participants in July and August 2019: four with providers who work in homeless health, three in reproductive health, and one in substance use treatment. Interviews averaged 27 min. As a result of the workshop, participants indicated that they modified the ways that they ask questions, had greater awareness of personal biases, and initiated conversations within their organizations about revising protocols.

Some participants discussed reflecting on and refining the questions they ask clients. One participant, a lactation consultant, reported that she changed how she inquired about previous children after learning that the topic of children could be triggering for clients who may have experienced forced child separation. Others spoke more generally about a greater sense of intentionality when engaging with clients as a result of the workshop. A case manager working in homelessness health said:

*Since our conversation at the workshop, I think it's been more on the back of my mind that when I'm asking a client for some information, am I asking it because I need that information, and it actually pertains, or do I not need that information, you know, for the activity that I need to do with this client?*

She felt that this reflection and self-evaluation “helped solidify the way in which [she] would treat another client with more dignity, with more mindfulness, or for their rights as a client.”

Others described how the workshop helped them reflect on their own personal biases. Three interview participants reflected on their responses to the pre-post questionnaire and how their biases changed after the workshop:

*There was a lot of questions about what do you think about homeless women who use [substances], who want to be pregnant. When I did [the pre-questionnaire], I was just like absolutely no. They should not. I think a lot of it wasn't coming from clients that I have worked with. I think it all came from personal bias. […] But, after hearing everyone speak, after going through the class, I thought that, you know, definitely my perspective has changed.*

Many participants identified the story-telling activity of the workshop as instrumental in their reflection process. The above participant further described the importance of stories: they “have a really big impact on people that maybe aren't familiar with working with that population or have a lot of biases to kind of see it and not just read about. It's different, and it's powerful.”

Finally, several participants also shared what they learned with their organizations in order to make a broader impact. One participant who works in homeless health engaged her coworkers about record keeping practices:

*I had good conversations with some colleagues around the questions piece […] When we're doing case notes, we're not over-indulging in terms of the narratives that we're creating for people. Don't make assumptions about things that you may be picking up on but really only document what happened.*

Another participant reported working with her supervisor, who also attended the workshop, to integrate anti-bias activities into future trainings within their organization.

## Discussion

This study demonstrates that a half-day training, developed in collaboration with a community and provider stakeholder group, resulted in decreased provider biases towards and increased provider confidence in addressing the reproductive health needs of women affected by homelessness and substance use. Key components of this workshop include its being developed in collaboration with a stakeholder group involving people with lived experience and inclusion of storytelling to increase providers’ empathy and decrease biases. Additionally, providers responded positively to having dedicated time to reflect in community with other providers and engage with intentional reflection on counseling approaches and the impact of standardized questions.

Provider bias in clinical decision making, particularly beliefs about who should or should not reproduce, can diminish reproductive autonomy and decrease healthcare utilization of marginalized and stigmatized groups. For example, providers may recommend and even pressure patients to use long-acting contraceptive methods because of judgment that, due to identity or circumstance, they should not become pregnant (Gomez et al., 2014; Holt et al., 2020). Studies have demonstrated that these patient-provider interactions can impact a variety of reproductive outcomes including future contraception use and birth experience (Altman et al., 2019; Dehlendorf et al., 2016). This holds true in the limited literature on unhoused cisgender women’s experiences accessing reproductive care, where, ultimately, biased treatment offered by providers creates barriers to entry and retention in care, including prenatal care and contraceptive services (Kennedy et al., 2014; Sznajder-Murray & Slesnick, 2011). Creating opportunities for providers to unpack their biases about the reproduction of people affected by homelessness and using substances and practice patient-centered care strategies may serve to uplift reproductive autonomy and encourage engagement in reproductive health services in diverse healthcare settings.

Centering the voices of those with lived experience in the development and execution of interventions serves to both challenge provider attitudes and ensure that interventions are relevant to the population being served (Julian et al., 2020). In this project, the stakeholder group set the workshop priorities, contributed intervention components, and reviewed and provided feedback on all components of the intervention. Including both individuals with lived experience with homelessness and substance involvement and providers who serve these communities as stakeholders in the process facilitated development of a nuanced intervention that prioritized affected women’s perspectives and respected the practical and emotional challenges of providers. The value of community-involved intervention development is reflected in our study results. Many participants stated that community perspectives and storytelling integrated into the training left the strongest impressions and may have had the greatest impact on provider attitudes. Involving providers, alongside individuals with lived experience, in our development process also allowed us to focus on key needs and intervention points uplifted by participants. Specifically, participants highlighted the importance of including intentional group time to reflect on and share uncertainties about serving patients with complex needs, and interrogating questions frequently asked in clinical encounters.

Interventions addressing patient-provider interactions are crucial and should be implemented together with actions to address structural barriers to care that impact person-centeredness. Our intervention addressed many areas desired by providers serving unhoused populations and people with substance use disorders, including resource sharing, networking, and a focus on empathy (Twis et al., 2021), as well as the priorities identified by our stakeholder group. However, after the workshop, participants reported feeling more overwhelmed by the complexity of the lives of affected clients. This finding is not surprising; there are few (if any) known interventions to address complex needs of this population in a time-limited setting. Interpersonal interventions, such as our project, should be integrated with structural changes to care delivery, both to improve patient-centered care and reduce provider burnout—a known result of providers’ feeling overwhelmed. For example, investing in gender-responsive, trauma-informed approaches to service delivery may both empower affected women to share their reproductive aspirations and feel heard and respected, while simultaneously improving support for providers (Covington et al., 2008; SAMHSA, 2014).

While the results of this evaluation show promise with respect to feasibility and short-term impact, our study had limitations. First, the workshop was conducted in San Francisco, where there has recently been an increase of health services focused on people experiencing homelessness and with substance use disorders. Our audience may have been primed to this topic and their receptivity may not be reflective of providers working in different contexts. Moreover, even within San Francisco, selection bias was likely at play as individuals elected (and were not required) to attend the workshop. Secondly, our community perspectives only included those of cisgender women. There is also, to our knowledge, no literature describing the reproductive aspirations and related healthcare needs of transgender people experiencing homelessness or using substances. Thus, while we used gender-neutral language during our pilot workshop, we did not account for the perspectives, or specific needs and challenges of transgender men and gender-expansive people at this intersection. We are not aware whether the workshop as framed would improve the care experience of these populations as they access reproductive health services.

Methodologically, our study included a small sample size, a lack of a control group, and attrition, which also limit transferability to other similar communities in different localities and contexts. In particular, individuals who did not complete the follow-up survey may have been less engaged in the workshop than those who chose to continue to participate, resulting in an over-estimation of impact. Results were based on self-report and could be exaggerated due to social desirability bias. Follow-up time was limited to one month, so we were unable to measure longer-term effects. Lastly, we did not directly measure provider behavior or how clients experienced counseling. Despite these limitations, this is one of the first attempts, to our knowledge, to create and measure the effects of a training specifically focused on reproductive health communication between providers and women experiencing homelessness and using substances. Our process was strengthened by the consistent contributions of community members through a patient-provider stakeholder group.

## Conclusions

Our results suggest that a half-day, community-informed workshop can reduce provider bias and increase confidence in counseling about reproductive health topics for providers working with women experiencing homelessness and using substances across a range of settings. Engaging relevant community stakeholders in the development and delivery of provider training may ensure relevance and increase impact.

## Data Availability

Data are not posted publicly. Qualitative transcripts contain professional details that could compromise the confidentiality of participants.

## Code Availability

Code for descriptive analyses can be made available by request.
